# Supplementary material for: Colocalized, bidirectional optogenetic modulations in freely behaving mice with a wireless dual-color optoelectronic probe
Source: Nat Commun. 2022 Feb 11;13:839. doi: 10.1038/s41467-022-28539-7 (PMC8837785; doi:10.1038/s41467-022-28539-7)
Supplement: Supplementary file 1 — Supplementary Information [file 41467_2022_28539_MOESM1_ESM.pdf]

Figure S1

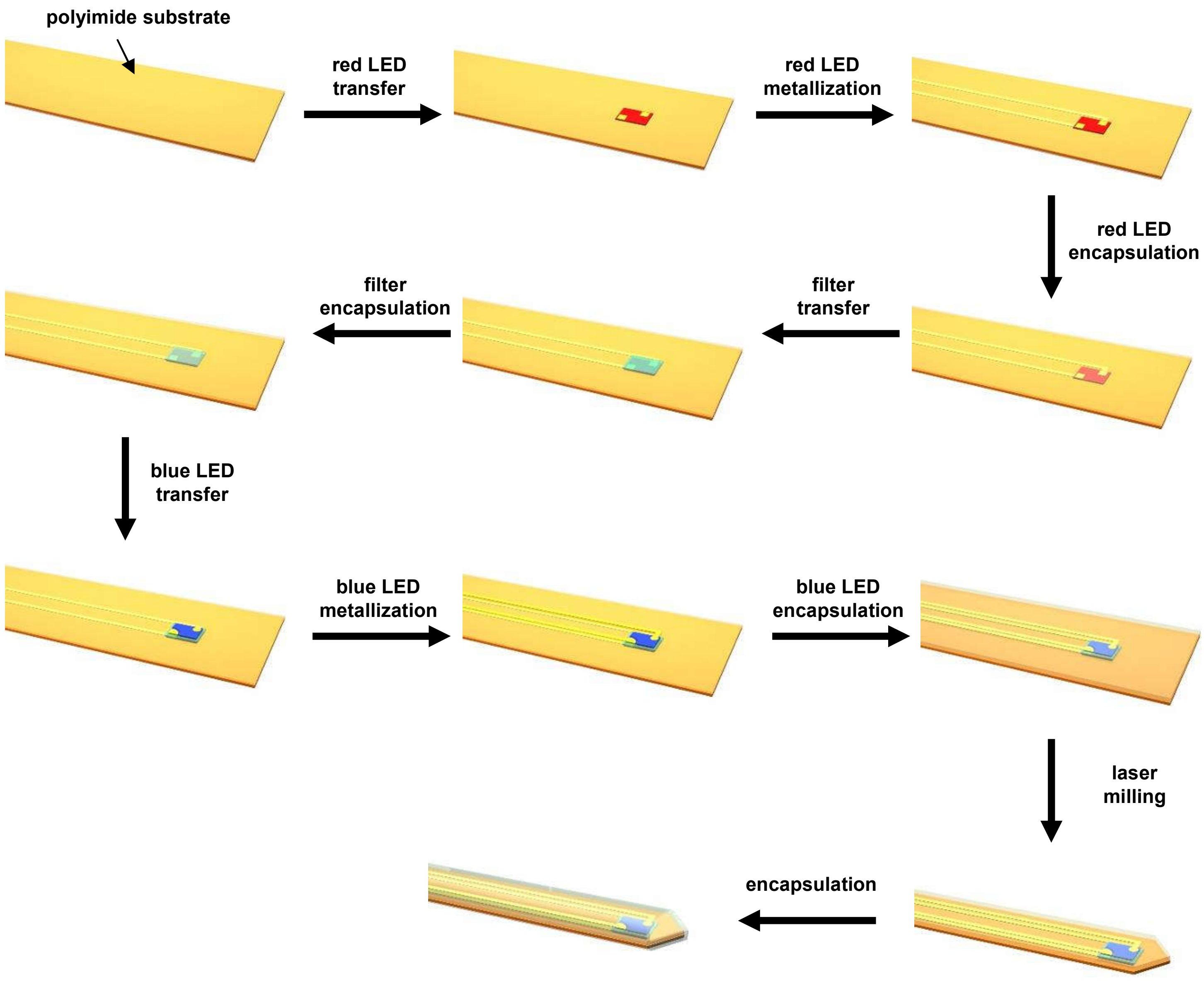

Figure S1. Schematic illustration of the process flow for fabricating the dual-color micro-LED probes, with vertically stacked red LED, filter and blue LED printed on PI substrates.

Figure S2

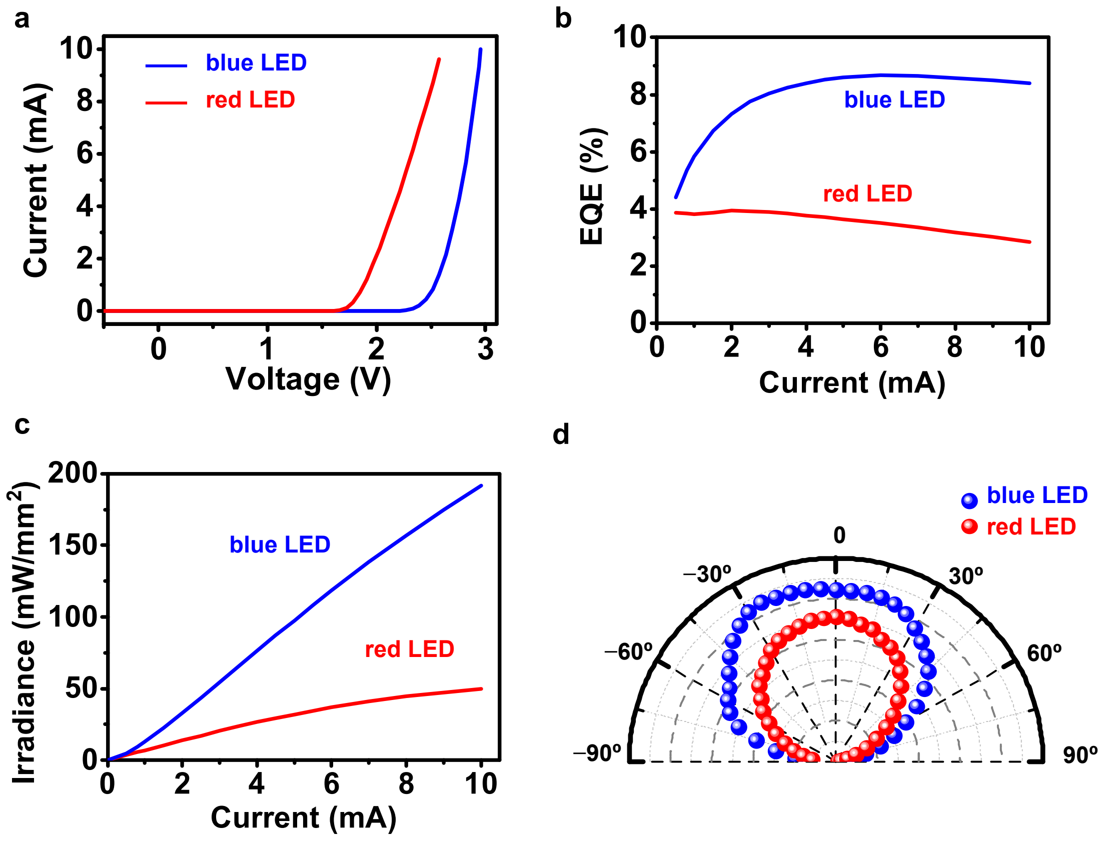

Figure S2. Measured optoelectronic properties of the red and the blue micro-LEDs in a probe structure. (a) Current–voltage curves. (b) External quantum efficiencies (EQEs) as a function of current. (c) Irradiance (power density on the LED surface) versus current. (d) Angular dependent emission profiles (in arbitrary unit). Source data are provided as a Source Data file.

Figure S3

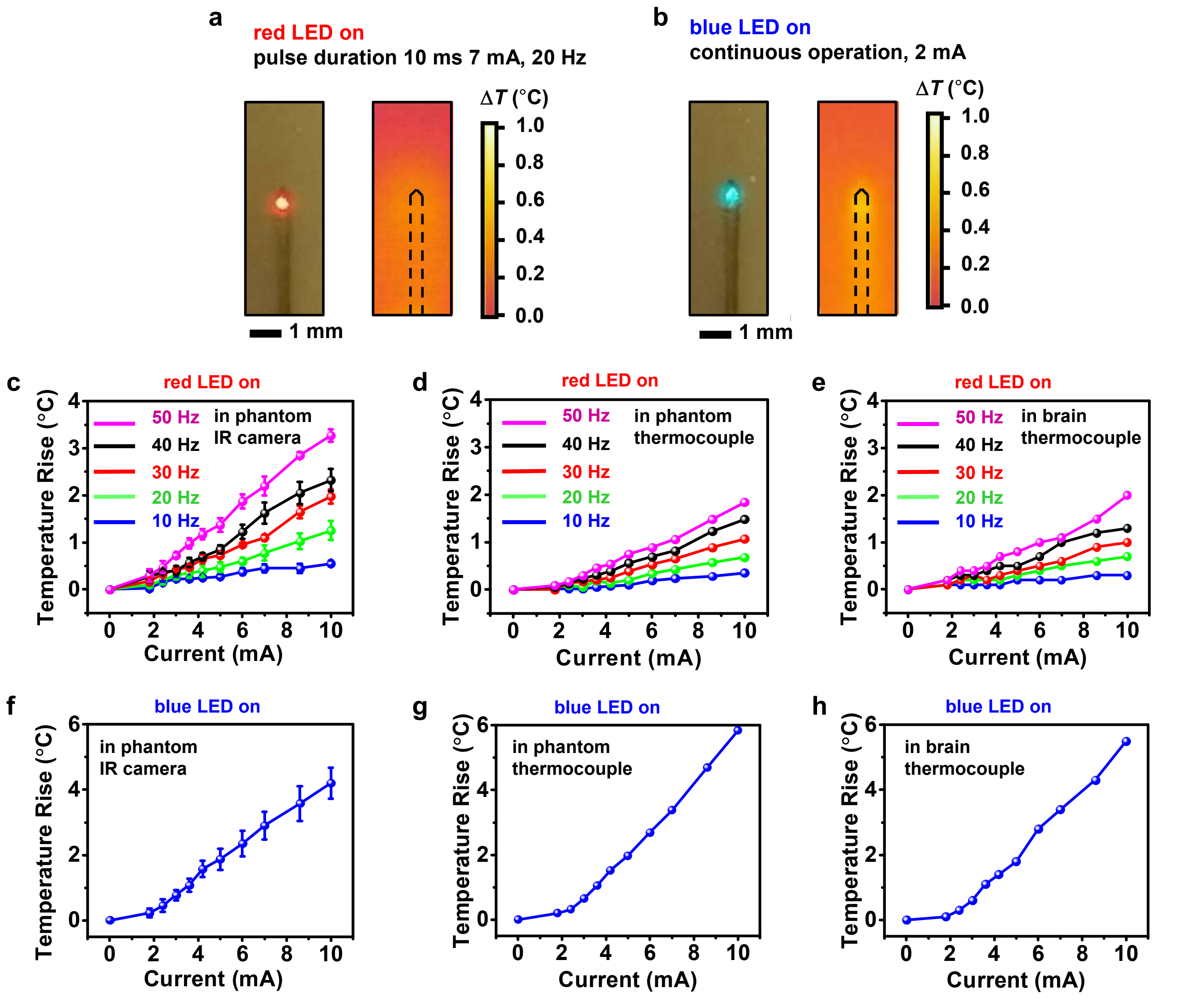

Figure S3. Measured thermal behaviors of a dual-color micro-LED probe. (a, b) Images and infrared (IR) thermographs showing a probe embedded into a brain phantom (~0.5 mm below the phantom surface). (a) Red LED on (injection current 7 mA, pulse frequency 20 Hz, pulse duration 10 ms). (b) Blue LED on (injection current 2 mA, continuous operation). (c) Measured temperature rise on the phantom surface by IR camera (n = 3 probes). (d) Measured temperature rise in the phantom by thermocouple. (e) Measured temperature rise in the brain of living mice by thermocouple. Red LED current 0–10 mA, frequency 10–50 Hz, pulse duration 10 ms). (f–h) Corresponding results when the blue LED is on (continuous mode, n = 3 probes in f). All data are represented as mean ± s.e.m.. Source data are provided as a Source Data file.

Figure S4

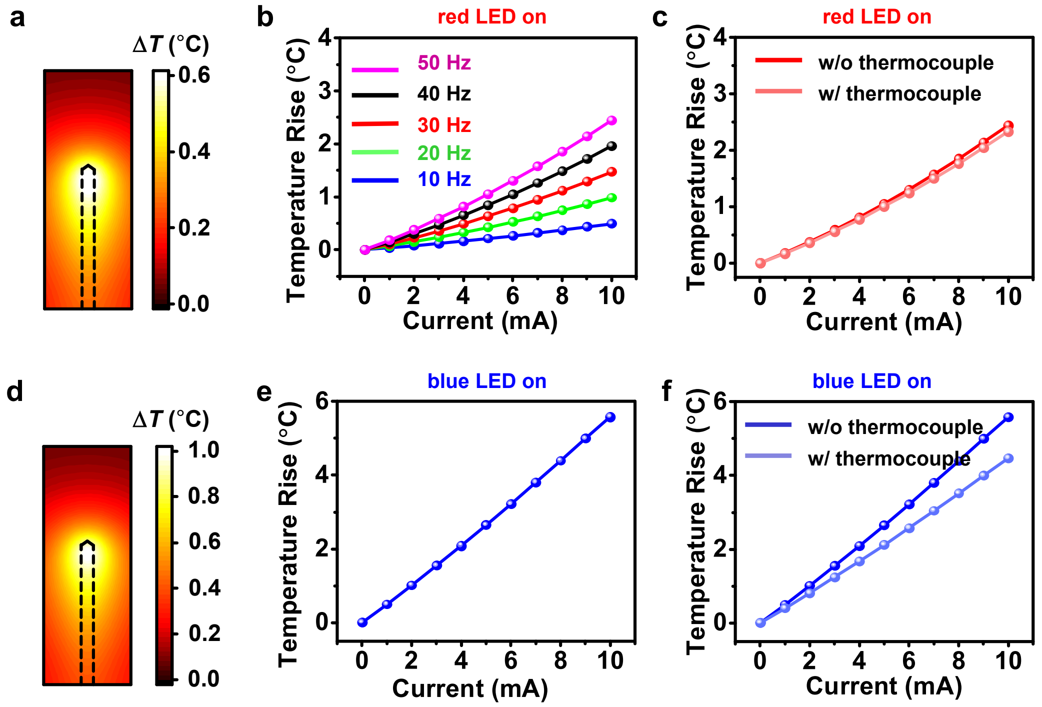

Figure S4. Simulated thermal behaviors of a dual-color micro-LED probe. (a) Simulated steady-state temperature distribution in the brain tissue surrounding the probe, when the red LED is on (current 7 mA, frequency 20 Hz, pulse duration 10 ms). (b) Simulated maximum temperature rise of the brain tissue when the red LED is in different operation conditions. (c) Simulated maximum temperature rise of the brain tissue when the red LED is on (frequency 50 Hz, pulse duration 10 ms). The results for models with and without the thermocouple are compared. (d–f) Corresponding results when the blue LED is on (continuous mode), current = 2 mA in (d). Source data are provided as a Source Data file.

Figure S5

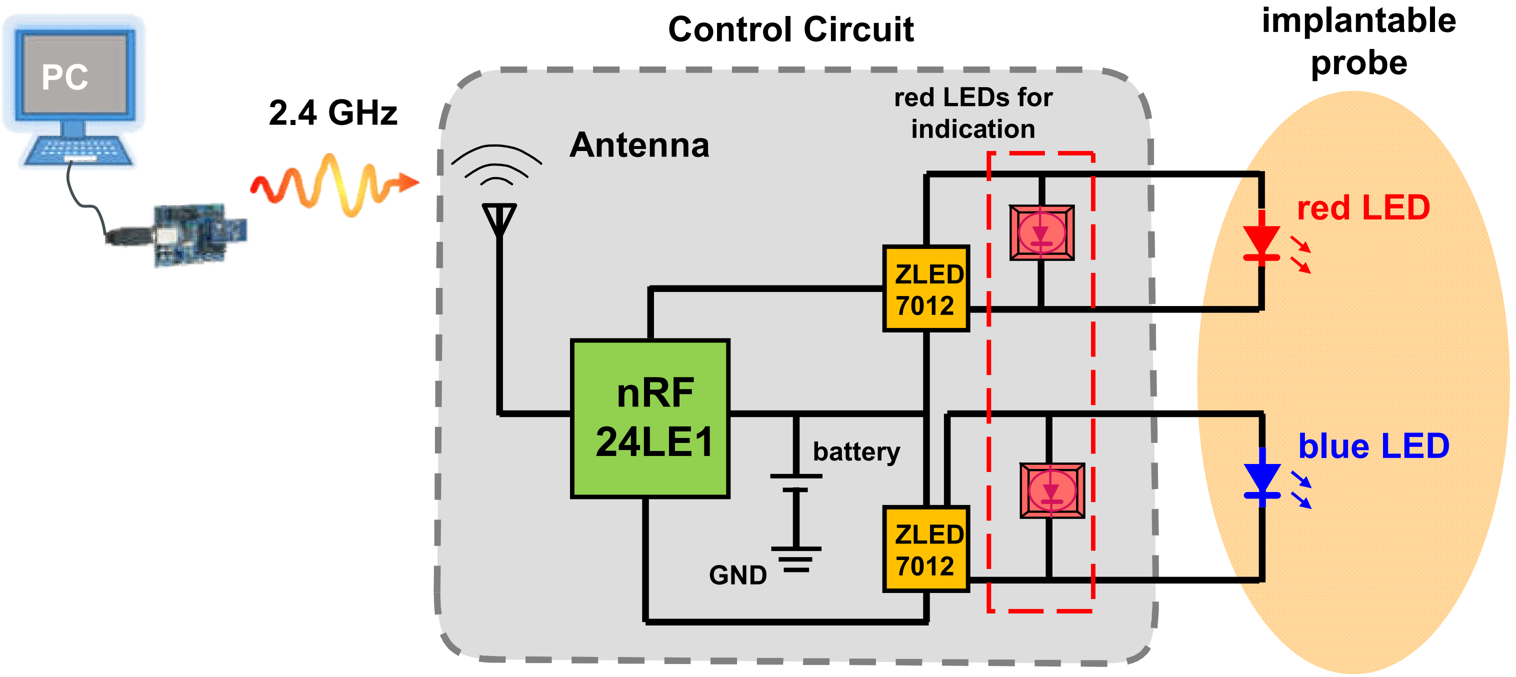

Figure S5. Schematic diagram showing the operational principle for the wireless circuit system controlling the dual-color micro-LED probe.

Figure S6

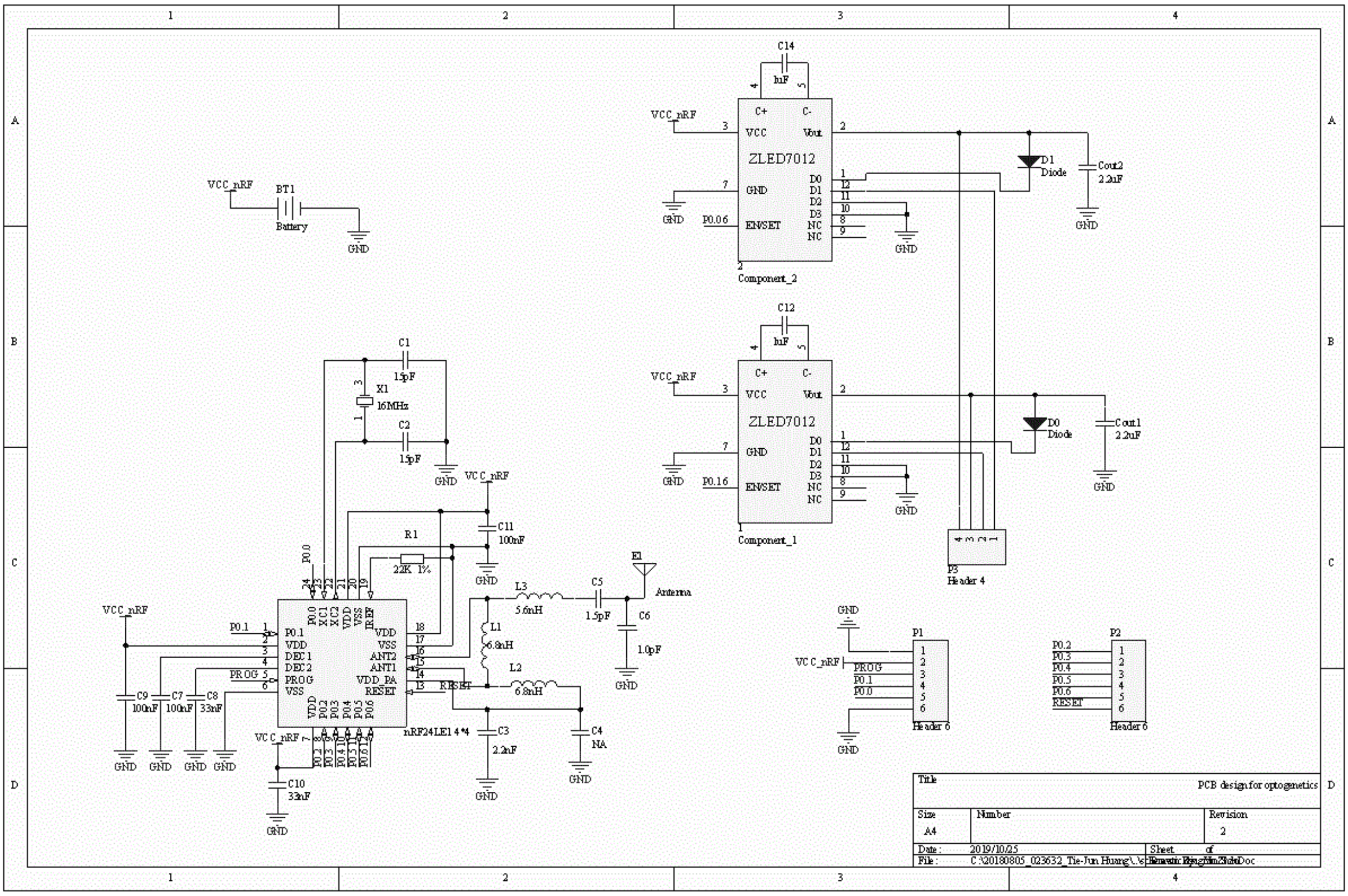

Figure S6. Detailed circuit design diagram of the wireless circuit system.

Figure S7

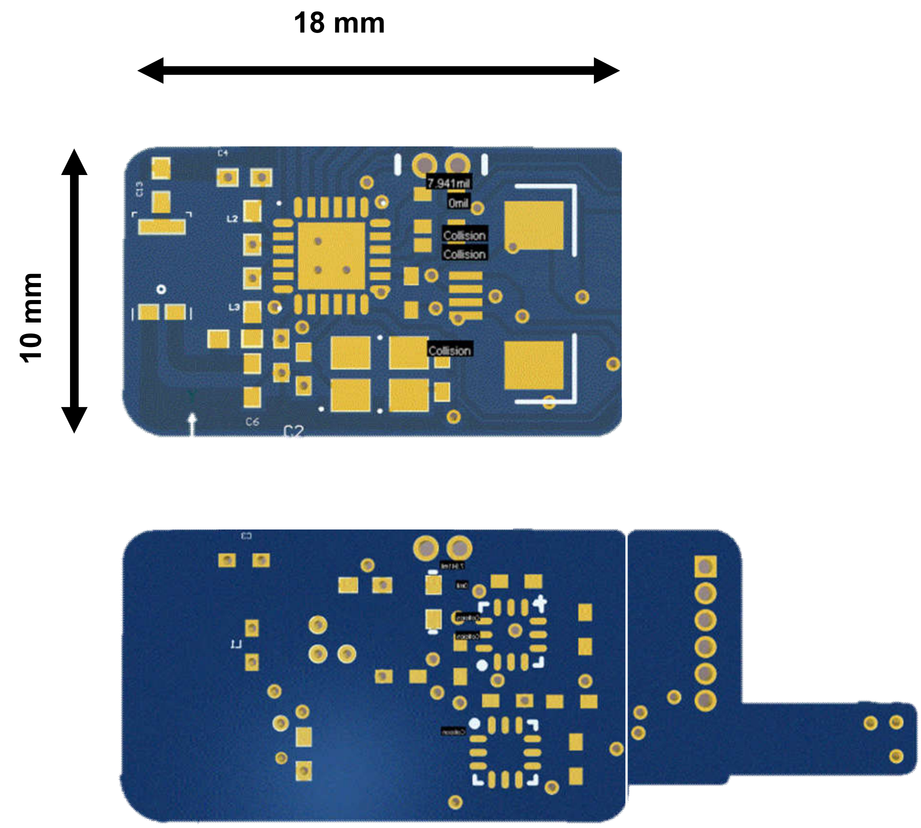

Figure S7. Layout of the designed printed circuit board (top: front view; bottom: back view).

Figure S8

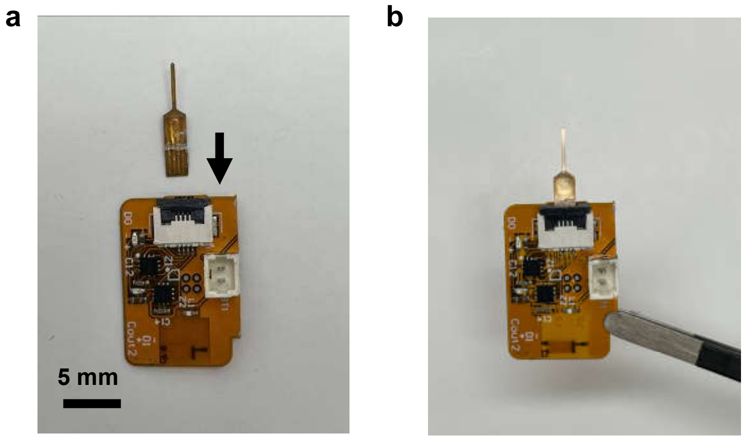

Figure S8. Optical images of a micro-LED probe and a control circuit (a) before and (b) after connection via a standard 4-pin connector.

Figure S9

	components
L1, L2	High frequency chip inductor +/- 5%, 6.8 nH
L3	High frequency chip inductor +/- 5%, 5.6 nH
C1, C2	NP0 +/- 2%, 15 pF
C3, C4	X7R +/- 10%, 2.2 nF
C5	NP0 +/- 0.1pF, 1.5 pF
C6	NP0 +/- 0.1pF, 1.0 pF
C7,C9,C11	X7R +/- 10%, 100 nF
C8,C10	X7R +/- 10%, 33 nF
C12,C14	X7R +/- 10%, 1 μF
Cout1,Cout2	2.2 μF
C13	NP0 50V, 10 pF
D0, D1	Indicating red LED, 610 nm, SMD 0402, 1 mm * 0.5 mm * 0.4 mm
R1	22 kΩ, 1%
nRF24LE1	nRF24LE1-F16Q24, QFN24 4 mm * 4 mm package, MCU
Component_1<br>Component_2	ZLED7012 ZI1R, LED current driver
X1	Crystal oscillator, SMD-3225, 16 MHz, CL=9 pF, +/- 60 ppm 3.2 mm * 2.5 mm package
E1	Antenna, H2U34WGTQW0100, AA055, 3.2 mm * 1.6 mm * 0.5 mm package
P1, P2	Pin Header, 6 pin, 1.25 mm, for programming
P3	FPC connector, 4 pin, 0.5 mm, for LED probe
P4	PCB header Connector, male, DIP Straight, 2 pin, 2.54 mm, for battery
Battery	Rechargeable lithium ion battery, 3.7 V, 45 mAh

Figure S9. List of components used in the wireless circuit system.

Figure S10

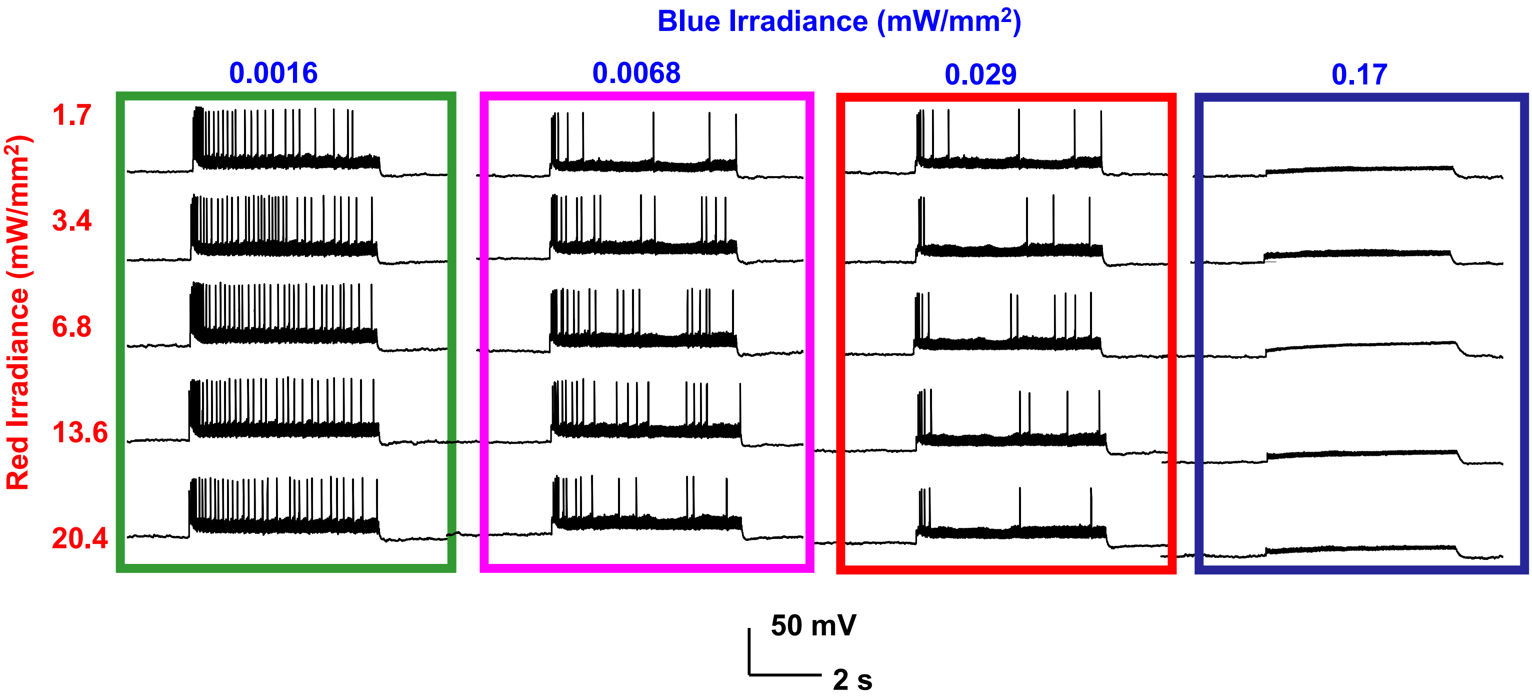

Figure S10. Recorded traces of a representative neuron with combined red and blue illumination at different irradiances. Red LED: 20 Hz, 10-ms pulse; Blue LED: continuous operation.

Figure S11

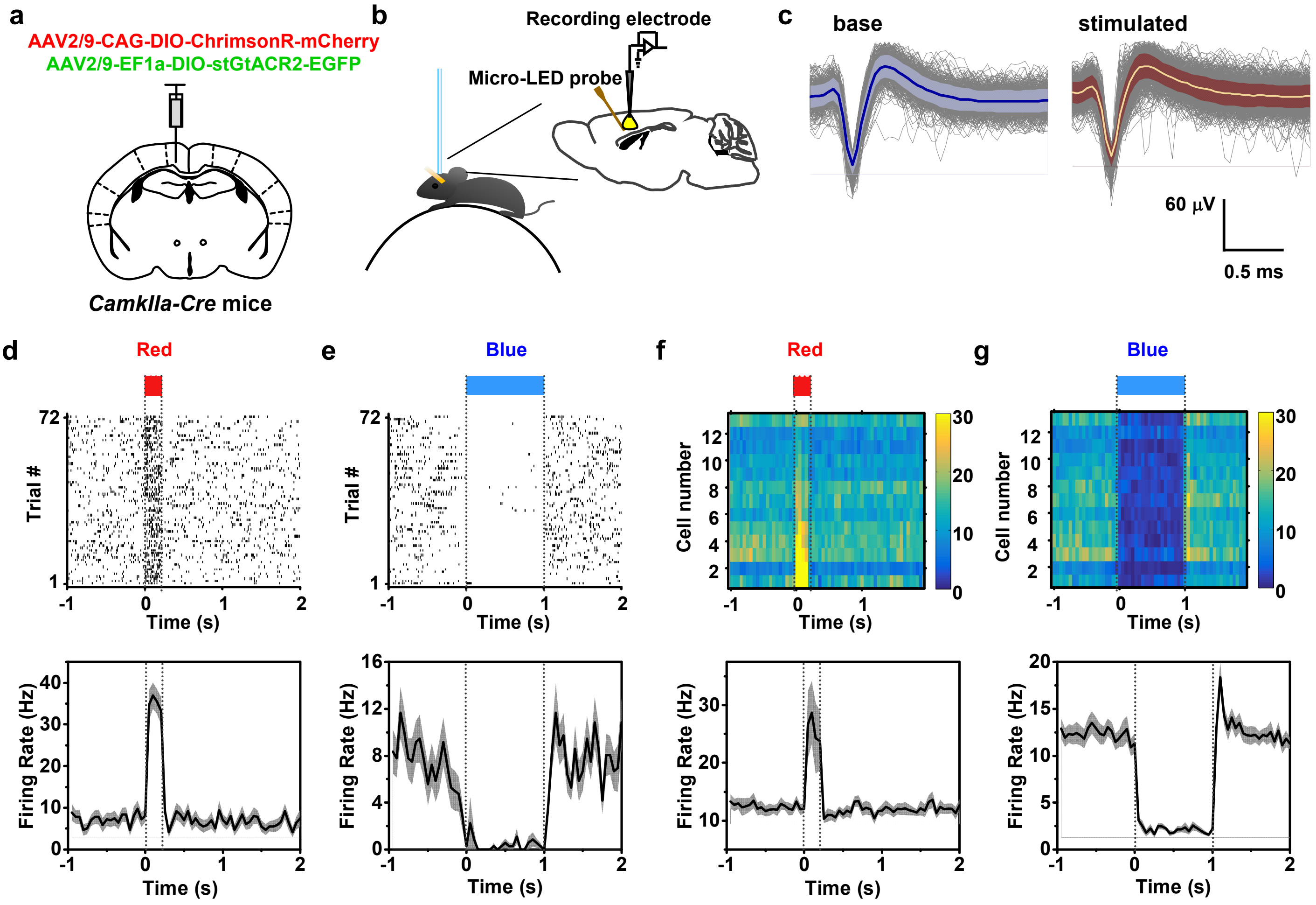

Figure 11. Bidirectional, *in vivo* optogenetic modulation of neural activities with dual-color illuminations in the cortex, combining with electrophysiological recordings. (a) Schematic strategy for co-expressing ChrimsonR and stGtACR2 in the primary somatosensory cortex of *CamkIIa-Cre* mice. (b) Illustration of the setup for simultaneous optogenetic stimulation and electrophysiological recordings by implanting the micro-LED probe and metal electrodes into the cortex of head-fixed mice. (c) Waveforms of a single unit recorded during baseline period before the red LED illumination (left) and during the red LED illumination period (right). Correlation coefficient between the two sets of waveforms is 0.9965. (d, e) Raster plots (top) and peri-stimulus time histogram (PSTH) plots (bottom) recorded for an example unit (n = 72 trials) for a sample unit during (d) red illumination (pulse width 0.2 s, LED current 10 mA) and (e) blue illumination (pulse width 1 s, LED current 1 mA). (f, g) Summarized results (top: heatmaps; bottom: PSTH plots) for multiple cells collected during (f) red illumination and (g) blue illumination (n = 13 cells from 2 mice, 50–100 trials for each cell). Shaded areas (d, e, f, g) indicate SEM. Source data are provided as a Source Data file.

Figure S12

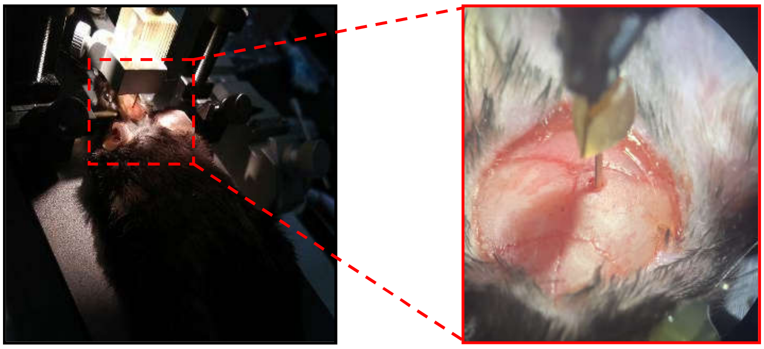

Figure S12. Photographs of the surgical procedure for probe implantation. A hole is created on the exposed skull of a mouse by drilling, and the dura is carefully removed by needle. The micro-LED probe is fixed on a holder controlled by a stereotaxic instrument, and slowly inserted into the targeted region.

Figure S13

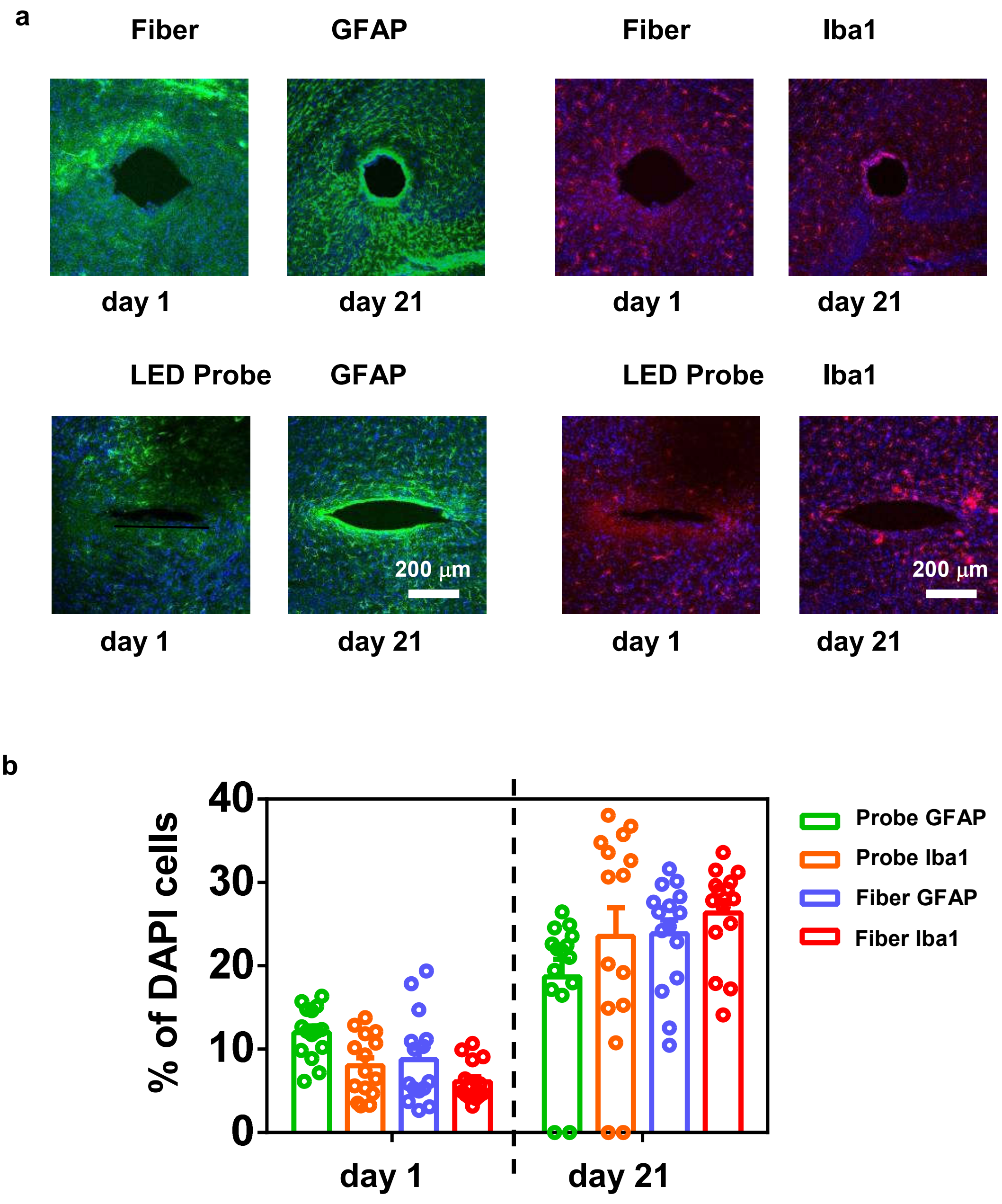

Figure S13. Immunoreactivity results showing the biocompatibility of the micro-LED probe, in comparison with those obtained with a silica based optical fiber. (a) Representative confocal fluorescence images of horizontal brain slices, showing immunohistochemical staining of astrocytes (GFAP) and activated microglia (Iba1) for both the LED probe and the fiber, after 1 day and 21 days implantation. Green: GFAP; Red: Iba1; Blue: DAPI. (b) Percentages of GFAP and Iba1 cell populations among DAPI cells collected at a distance of 200 μm from the edge of implantation. The LED probe and the fiber show similar results of inflammatory glial responses occurring after implantation. (n = 3 mice for each group, and 5 slices for each mice). All data are represented as mean ± s.e.m.. Source data are provided as a Source Data file.

Figure S14

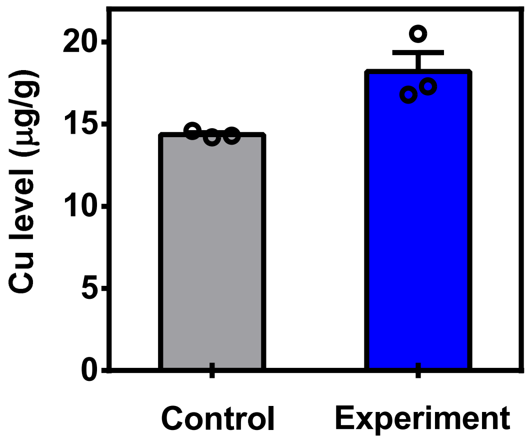

Figure S14. Measured Cu concentration in the brain tissue. The brain tissue is dried and analyzed with inductively coupled plasma mass spectrometry (Thermo ICP-MS iCAPQ, ThermoFisher, USA). The measured Cu level is 18.2 ± 2.3 µg/g for mice 5 weeks post probe implantation (experiment group). The result for the control group without probe is 14.4 ± 0.2 µg/g. The Cu levels in both cases are in the normal range (< 20 µg/g in mice brain[1], and 30–100 µg/g in human brain[2]). *n* = 3 mice for each group. All data are represented as mean ± s.e.m.. Source data are provided as a Source Data file.

Figure S15

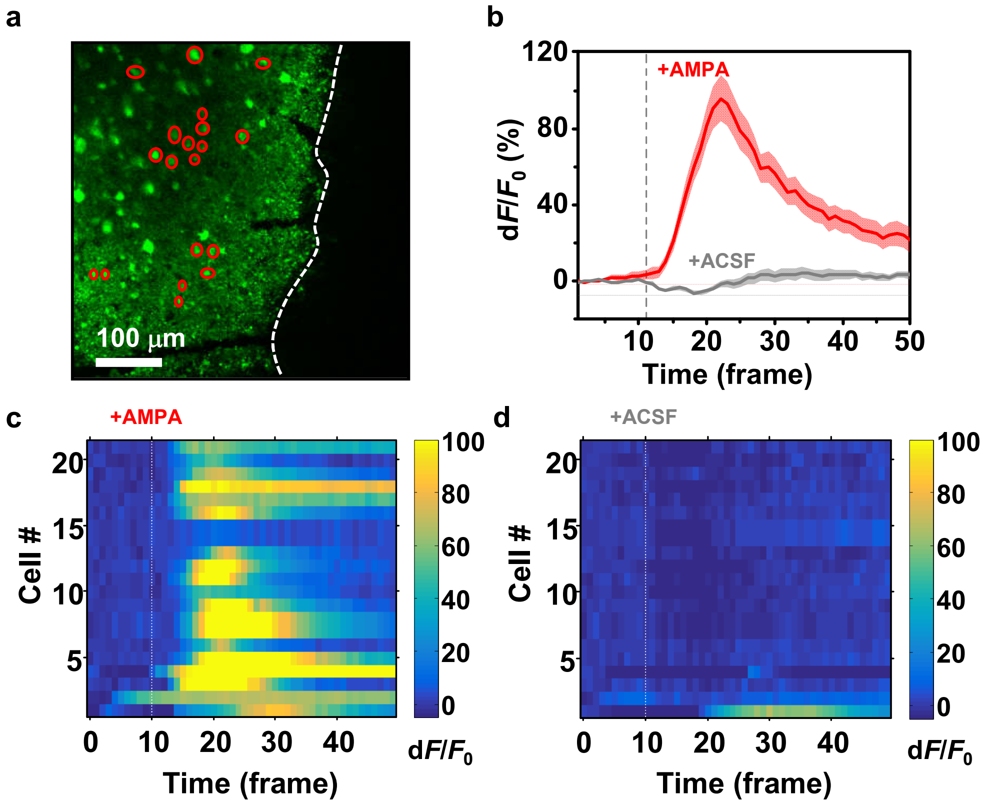

Figure S15. Imaging cellular calcium dynamics in acute brain slice. (a) Representative fluorescence image showing GCaMP6m-expressing cells near the probe region after 14 days implantation. Red circles mark active cells followed by AMPA administration. The dashed line marks the border of the lesion area. (b) Averaged calcium signal traces (dF/F_0) for all marked neurons after applying AMPA (red line) or pure ACSF (grey line) at frame 11, the sampling rate is 1.1 s / frame. Shaded areas indicate SEM. (c, d) Heatmaps showing fluorescence variations in all 21 neurons after (c) applying AMPA or (d) pure ACSF.

Figure S16

a

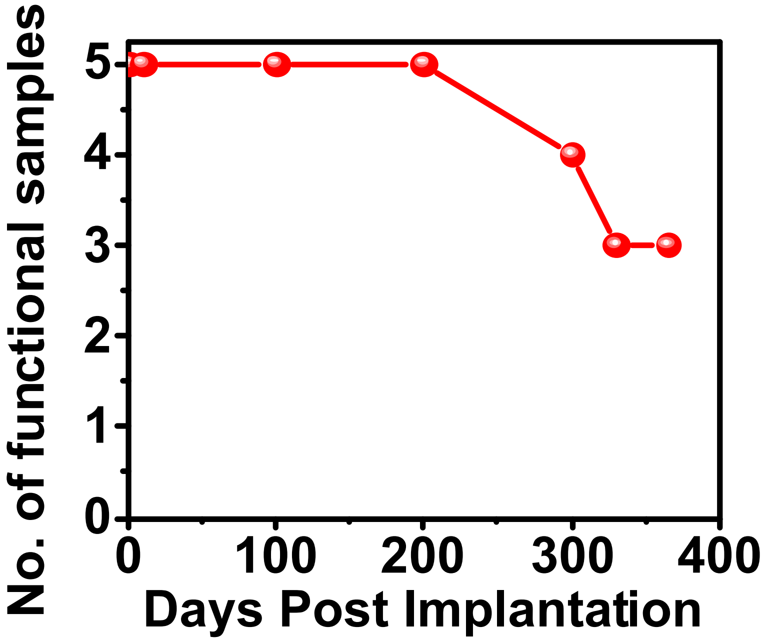

b

Mouse # / Date	Forward voltage (at current = 0.7 mA)	2019.09.05 (1 day)	2019.12.15 (100 days)	2020.03.25 (200 days)	2020.07.05 (300 days)	2020.08.05 (330 days)	2020.09.05 (365 days)
1	Red	1.81	1.82	1.81	1.83	1.83	1.83
	Blue	2.47	2.46	2.47	2.47	2.47	2.47
2	Red	1.83	1.83	1.83	1.83	1.85	1.85
	Blue	2.47	2.47	2.48	> 20 (broken)	-	-
3	Red	1.83	1.83	1.83	1.83	animal died	-
	Blue	2.47	2.47	2.47	2.47	animal died	-
4	Red	1.83	1.83	1.83	1.83	1.82	1.82
	Blue	2.47	2.47	2.47	2.47	2.46	2.46
5	Red	1.83	1.83	1.83	1.83	1.83	1.83
	Blue	2.46	2.46	2.47	2.46	2.46	2.46

Figure S16. (a) Chronic stability for the dual-color micro-LED probe. 5 probes are separately implanted into 5 behaving mice. Probes with both red and blue micro-LEDs operating in the normal condition are defined as "functional probes". (b) Summary of the performance for red and blue micro-LEDs in each probe. These probes are kept within the mouse brain. Their performance are evaluated by measuring LED's forward voltages at a constant current of 0.7 mA. After 365 days, the functional probes are taken out and still operate normally.

Figure S17

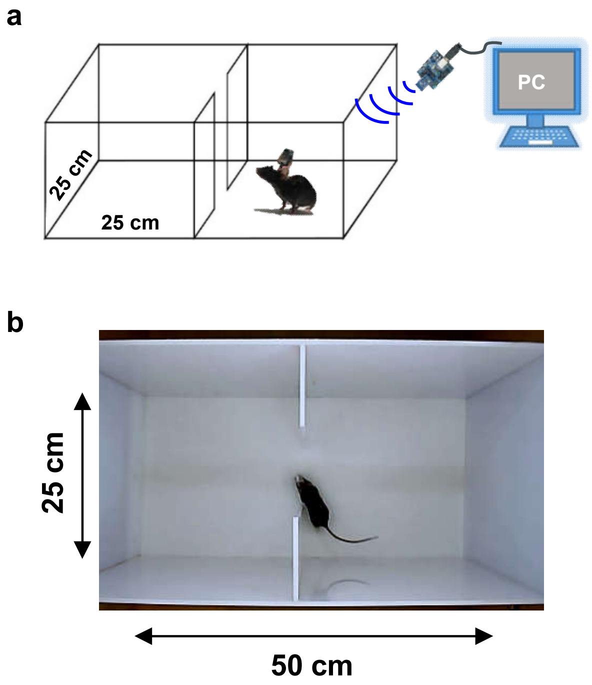

Figure S17. (a) Schematic diagram and (b) top-view photograph of the real-time place preference test with a two-compartment arena.

Figure S18

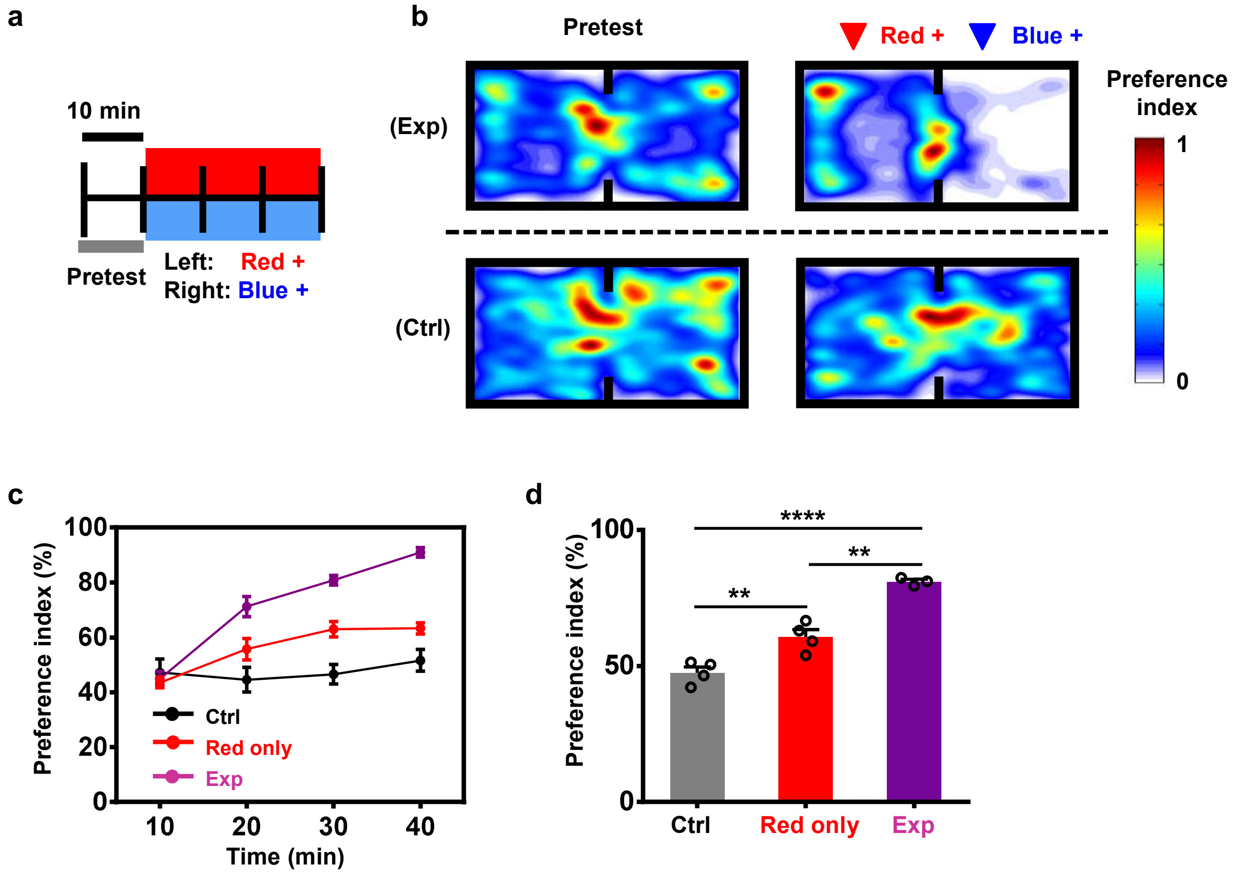

Figure S18. (a) Patterns used for optogenetic modulations, including a 10-min pretest, a 30-min red LED stimulation (20 Hz, 10-ms pulse, current 7 mA) in the left chamber and blue LED stimulation (continuous, current 5 mA) in the right chamber. (b) Representative heat maps comparing pretest and real-time preference behavior following both red (left chamber) and blue (right chamber) stimulation for mice expressing stGtACR2 + ChrimsonR (experiment group) and EGFP + mCherry (control group). (c) Preference indices measured at different times for mice under only red stimulations (red line, $n = 4$ mice), or red stimulations in the left chamber and blue stimulations in the right chamber (exp, purple line, $n = 3$ mice), and the control group with the red stimulation in the left chamber and blue light in the right chamber (ctrl, black line, $n = 4$ mice). (d) Summary of preference indices (the ratio of the time that mice spend in the left chamber to the whole recorded time) for mice under only red stimulations ($n = 4$ mice), or red stimulations in the left chamber and blue stimulations in the right chamber ($n = 3$ mice) for both experiment and control groups. Two-tailed unpaired *t* test, ** $P < 0.01$, **** $P < 0.0001$. All data are represented as mean ± s.e.m.. See Supplementary Table 1 for detailed statistical analysis. Source data are provided as a Source Data file.

Figure S19

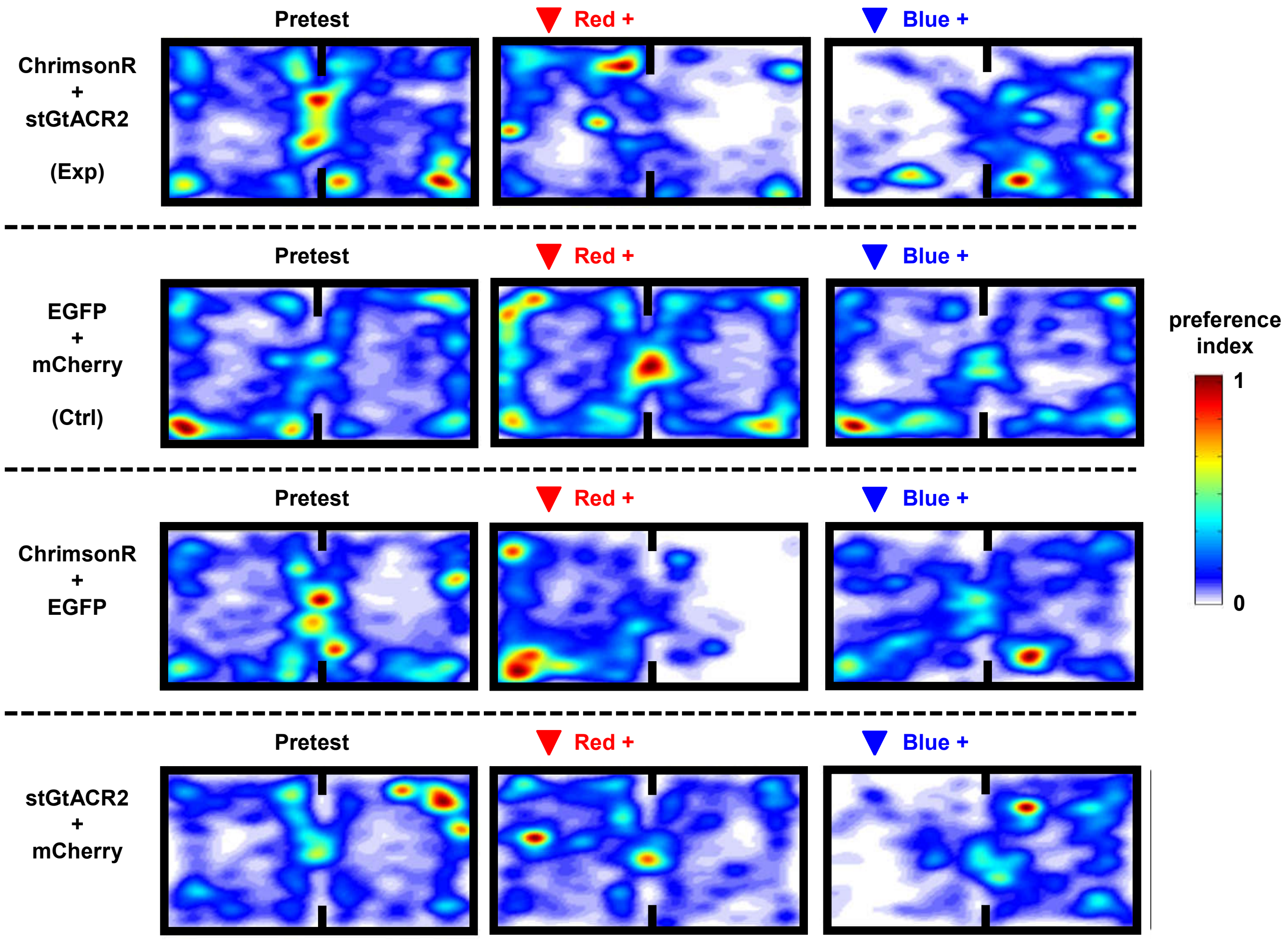

Figure S19. Representative heat maps showing real-time preference and aversion behavior following red or blue stimulation for mice expressing stGtACR2 + ChrimsonR (experiment, or Exp), EGFP + mCherry (control, or Ctrl), ChrimsonR + EGFP and stGtACR2 + mCherry. The data for ChrimsonR + stGtACR2 and EGFP + mCherry groups are identical to those presented in Fig. 3e.

Figure S20

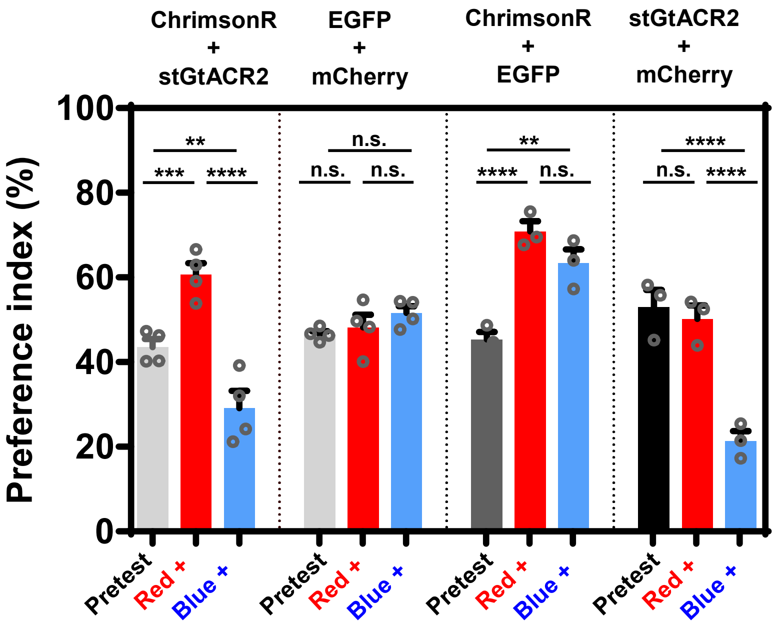

Figure S20. Summary of preference indices (the ratio of the time that mice spend in the left chamber to the whole recorded time) under red and blue stimulations for different mouse groups expressing ChrimsonR + stGtACR2 (n = 4 mice), EGFP + mCherry (n = 4 mice), ChrimsonR + EGFP (n = 3 mice), stGtACR2 + mCherry (n = 3 mice). Student's t test, ** $P < 0.01$, *** $P < 0.001$, n.s. $P > 0.05$. The data for ChrimsonR + stGtACR2 and EGFP + mCherry groups are identical to those presented in Fig. 3f. Two-way ANOVA, Sidak's multiple-comparisons test (pretest versus stimulations). All data are represented as mean ± s.e.m.. See Supplementary Table 1 for detailed statistical analysis. Source data are provided as a Source Data file.

Figure S21

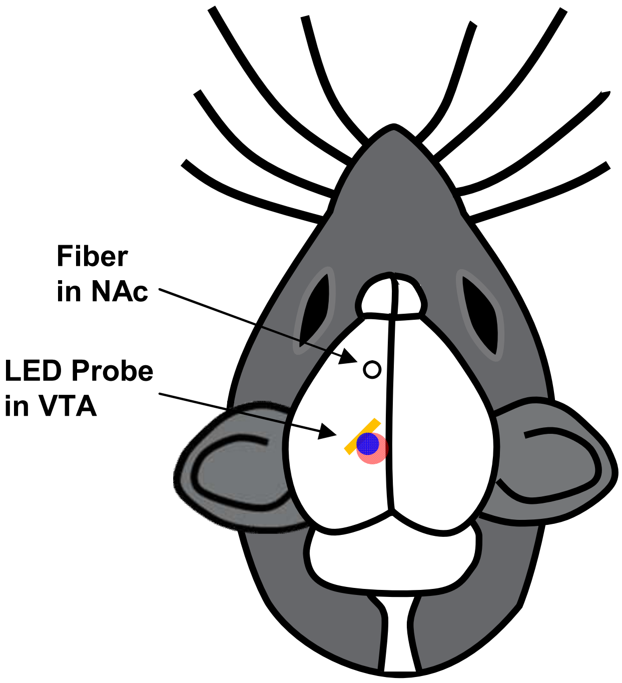

Figure S21. Schematic illustration (horizontal view) for the implantation of the fiber for DA recording in the NAc, and the dual-color LED probe for optogenetic stimulations in the VTA. The specific insertion angle is chosen for the LED probe, to minimize the optical crosstalk that disturbs the fiber photometric recording in the NAc.

Figure S22

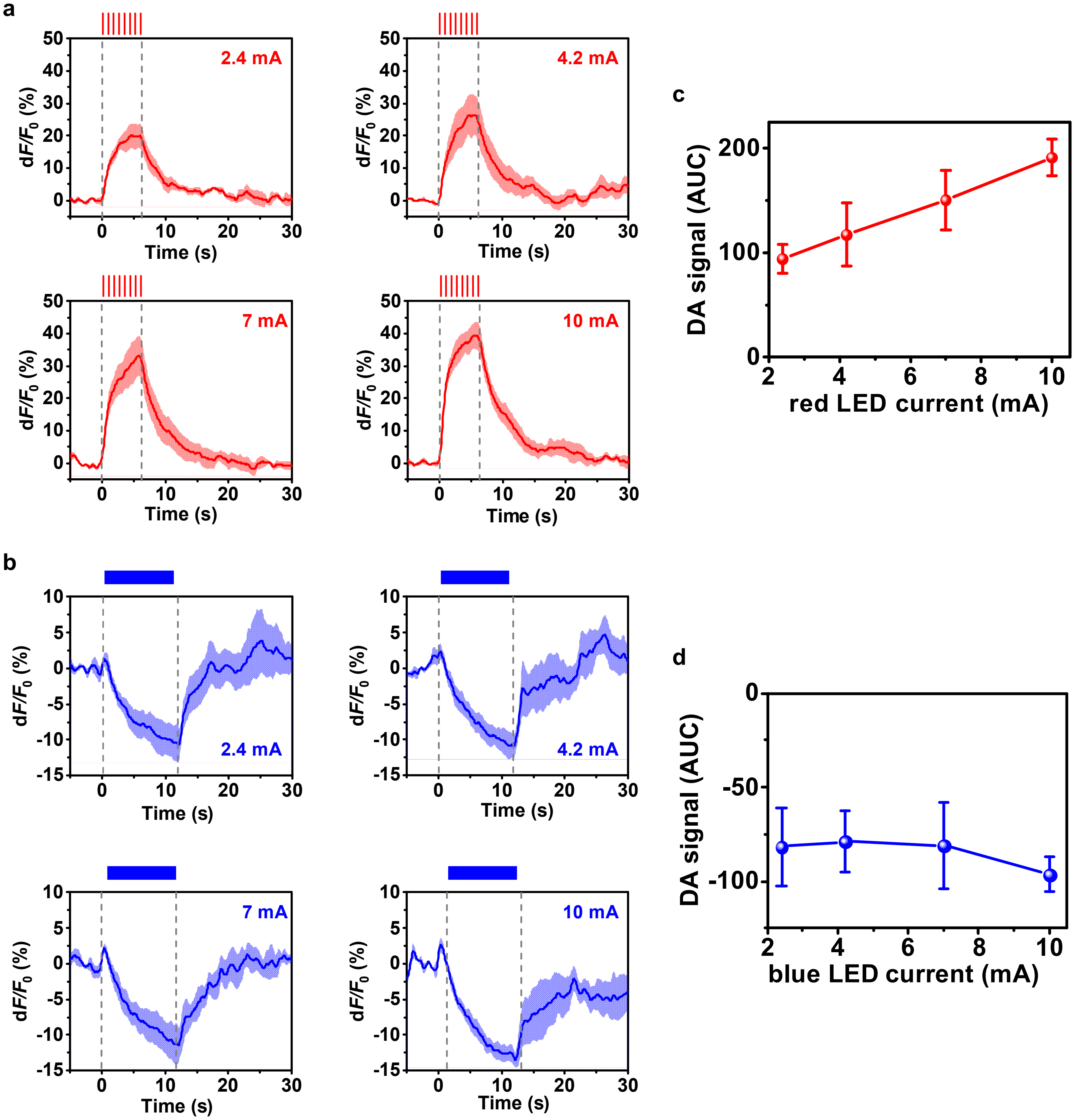

Figure S22. DA signals recorded as the fluorescence of $GRAB_{DA2m}$ in the NAc, under optogenetic stimulations applied in the VTA by red or blue micro-LEDs. (a, b) Representative traces of DA signals with stimulations by (a) the red LED (20 Hz, 20 ms, 6 s, $n = 4$) and (b) the blue LED (continuous, 12 s, $n = 4$ mice) at various currents. The solid lines and shaded areas indicate the mean and s.e.m., respectively ($n = 4$ mice). (c, d) Accumulative DA signals with (c) red ($n = 4$ mice) and (d) blue stimulations ($n = 4$ mice) under different LED currents. AUC: area under the curve during stimulation. The data for red LED at 7 mA and blue LED at 2.4 mA, and (c), (d) are identical to those presented in Figure 4c–4e. All data are represented as mean ± s.e.m.. Source data are provided as a Source Data file.

Figure S23

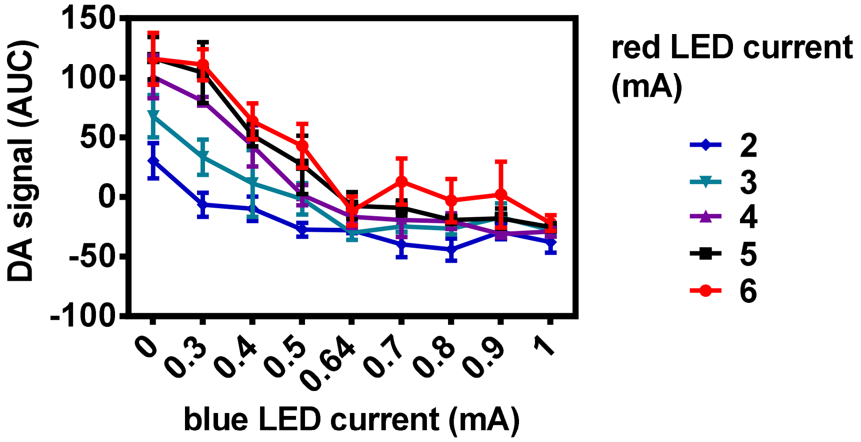

Figure S23. Detailed results of measured DA signals (AUC) with the combination of red and blue irradiance at different LED currents simultaneously for 6 s (*n* = 4 mice). All data are represented as mean ± s.e.m.. Source data are provided as a Source Data file.

Figure S24

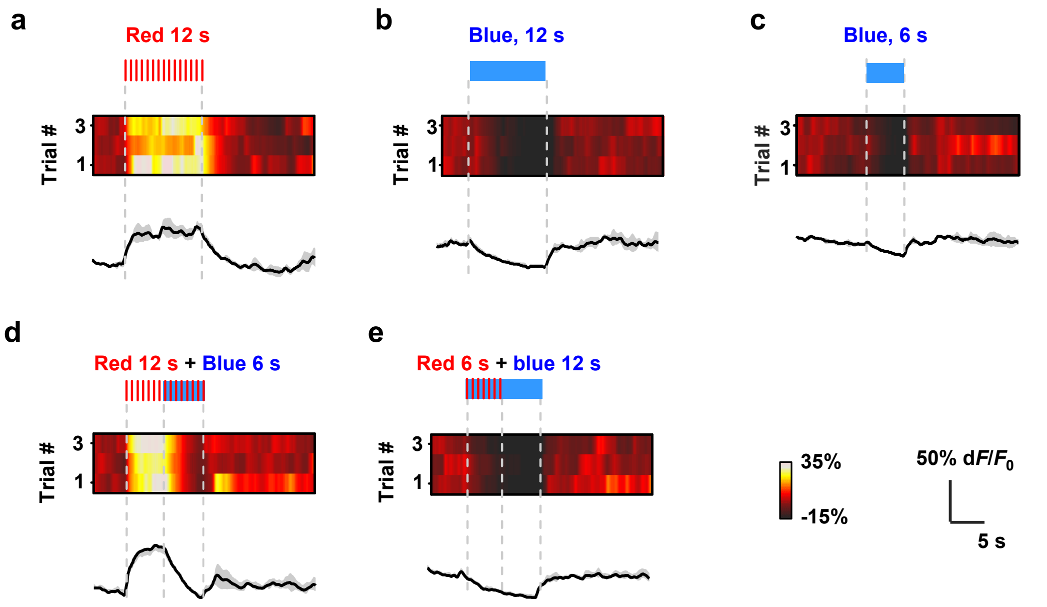

Figure S24. Example traces of optogenetically evoked DA transients for mice co-expressing stGtACR2 + ChrimsonR, in response to different stimulation patterns. (a) Red LED on, 20 Hz, 20-ms pulse, 10 mA, 0–12 s. (b) Blue LED on, continuous, 3 mA, 0–12 s. (c) Blue LED on, continuous, 3 mA, 6–12 s. (d) Red LED on 0–12 s and Blue LED on 6–12 s. (e) Red LED on 0–6 s and Blue LED on 0–12 s. The solid lines and shaded areas indicate the mean and s.e.m., respectively.

Table S1

Figure #	Analysis	Conditions	significance	*P* value
Fig. 3f	Two-way ANOVA	Exp		
		pretest vs. red	**	0.0023
		pretest vs. blue	**	0.0081
		red vs. blue	****	< 0.0001
		Ctrl		
		pretest vs. red	n.s.	0.9651
		pretest vs. blue	n.s.	0.5082
		red vs. blue	n.s.	0.774
Fig. 4c	Two-way ANOVA	Exp-red vs. Exp-blue	**	0.0035
		Exp-red vs. Ctrl-red	*	0.0149
		Exp-blue vs. Ctrl-blue	*	0.0131
		Ctrl-red vs. Ctrl-blue	n.s.	0.998
Fig. 4f	Two-way ANOVA	Exp-A		
		red-1 vs. red-2	n.s.	0.9131
		red-1 vs. blue	*	0.0364
		red-2 vs. blue	*	0.0463
		Exp-B		
		red-1 vs. blue	****	< 0.0001
		red-1 vs. red-3	n.s.	0.455
		red-3 vs. blue	****	< 0.0001
		Exp-C		
		blue vs. red-2	n.s.	0.0935
		blue vs. red-3	*	0.0294
		red-2 vs. red-3	n.s.	0.5883
		Ctrl-A		
		red-1 vs. red-2	n.s.	0.8194
		red-1 vs. blue	n.s.	0.3749
		red-2 vs. blue	n.s.	0.2666
		Ctrl-B		
		red-1 vs. blue	n.s.	0.6327
		red-1 vs. red-3	n.s.	0.5264
		red-3 vs. blue	n.s.	0.8756
		Ctrl-C		
		blue vs. red-2	n.s.	0.6116
		blue vs. red-3	n.s.	0.4368
		red-2 vs. red-3	n.s.	0.7857

Table S1. Summary of statistical analyses.

Table S1 (continued)

Figure #	Analysis	Conditions	significance	*P* value
Fig. 5e	Two-way ANOVA	red vs. blue		
		ChrimsonR+stGtACR2	****	< 0.0001
		ChrimsonR	****	< 0.0001
		stGtACR2	**	0.0011
		Ctrl	n.s.	0.9996
Fig. S18d	Two-tailed unpaired *t* test	Red only vs. Ctrl	**	0.009
		Exp vs. Red only	**	0.0016
		Exp vs. Ctrl	****	< 0.0001
Fig. S20	Two-way ANOVA	ChrimsonR + stGtACR2		
		pretest vs. red	***	0.0007
		pretest vs. blue	**	0.0035
		red vs. blue	****	< 0.0001
		EGFP + mCherry		
		pretest vs. red	n.s.	0.9635
		pretest vs. blue	n.s.	0.4846
		red vs. blue	n.s.	0.7629
		ChrimsonR + EGFP		
		pretest vs. red	****	< 0.0001
		pretest vs. blue	**	0.0017
		red vs. blue	n.s.	0.2702
		stGtACR2 + mCherry		
		pretest vs. red	n.s.	0.8933
		pretest vs. blue	****	< 0.0001
		red vs. blue	****	< 0.0001

Table S1 (continued). Summary of statistical analyses.

References

1. Chen Y, Wang L, Geng J-H, Zhang H-F, Guo L. Apolipoprotein E deletion has no effect on copper-induced oxidative stress in the mice brain. Biosci Rep 2018, 38(5).

2. Harrison WW, Netsky MG, Brown MD. Trace elements in human brain: Copper, zinc, iron, and magnesium. Clinica Chimica Acta 1968, 21(1): 55-60.
