# Supplementary material for: Colocalized, bidirectional optogenetic modulations in freely behaving mice with a wireless dual-color optoelectronic probe
Source: Nat Commun. 2022 Feb 11;13:839. doi: 10.1038/s41467-022-28539-7 (PMC8837785; doi:10.1038/s41467-022-28539-7)
Supplement: Supplementary file 3 — Description of Additional Supplementary Files [file 41467_2022_28539_MOESM3_ESM.pdf]

Title: Supplementary Movie 1.
Description: Video for dual-color micro-LED probe, displaying alternating blue and red emissions.

Title: Supplementary Movie 2.
Description: Video for multiple dualcolor LED probes assembled with wirelessly operated circuit modules, showing capabilities for independent light emission control.

Title: Supplementary Movie 3.
Description: Video for four freely moving mice implanted with micro-LED probes and headmounted circuits.

Title: Supplementary Movie 4.
Description: Video for multi-channel dual-color probes with 6 pairs of stacked red-blue micro-LEDs, displaying alternating blue and red emissions. These micro-LEDs are driven by a wired external power source.

Title: Supplementary Movie 5.
Description: Video for a tri-color (redgreen-blue) micro-LED needle structure, displaying different colors under current injection. These micro-LEDs are driven by a wired external power source
